# Mapping gene expression quantitative trait loci by singular value decomposition and independent component analysis

**DOI:** 10.1186/1471-2105-9-244

**Published:** 2008-05-20

**Authors:** Shameek Biswas, John D Storey, Joshua M Akey

**Affiliations:** 1 Department of Genome Sciences, University of Washington, 1705 NE Pacific Street, Seattle, WA, 98195, USA; 2 Department of Biostatistics, University of Washington, 1705 NE Pacific Street, Seattle, WA, 98195, USA

## Abstract

**Background:**

The combination of gene expression profiling with linkage analysis has become a powerful paradigm for mapping gene expression quantitative trait loci (eQTL). To date, most studies have searched for eQTL by analyzing gene expression traits one at a time. As thousands of expression traits are typically analyzed, this can reduce power because of the need to correct for the number of hypothesis tests performed. In addition, gene expression traits exhibit a complex correlation structure, which is ignored when analyzing traits individually.

**Results:**

To address these issues, we applied two different multivariate dimension reduction techniques, the Singular Value Decomposition (SVD) and Independent Component Analysis (ICA) to gene expression traits derived from a cross between two strains of *Saccharomyces cerevisiae*. Both methods decompose the data into a set of meta-traits, which are linear combinations of all the expression traits. The meta-traits were enriched for several Gene Ontology categories including metabolic pathways, stress response, RNA processing, ion transport, retro-transposition and telomeric maintenance. Genome-wide linkage analysis was performed on the top 20 meta-traits from both techniques. In total, 21 eQTL were found, of which 11 are novel. Interestingly, both *cis *and *trans*-linkages to the meta-traits were observed.

**Conclusion:**

These results demonstrate that dimension reduction methods are a useful and complementary approach for probing the genetic architecture of gene expression variation.

## Background

Recently, the combination of gene expression profiling and classic quantitative trait locus (QTL) mapping has emerged as an important tool in dissecting the genetic basis of gene expression variation [1-5]. Using transcript levels as surrogates for higher order quantitative traits enables a finer scale resolution of the underlying molecular basis of complex phenotypes. Gene expression traits can also be integrated with network inference methods to reconstruct genetic pathways and metabolic networks from genetic perturbation data [6,7]. The first expression QTL (eQTL) study was performed in offspring derived from a cross between two divergent strains of *Saccharomyces cerevisiae *[1]. Linkage analyses revealed thousands of eQTLs, acting both in *cis *and *trans*, with most *trans*-linkages being due to a few regulatory "hotspots".

Current statistical methods that analyze high-dimensional phenotypes, such as expression traits, one trait at a time suffer from low power because of the challenges associated with multiple hypothesis testing. In addition, such approaches fail to take advantage of the potentially informative correlation structure of high-dimensional phenotypes. In order to exploit the correlation structure among genes, various data reduction techniques can be used to reduce the overall dimensionality of the data. For example, in the context of eQTL studies, hierarchical clustering has been performed followed by linkage mapping of the average expression of cluster members [8]. Such an approach is constrained by the size and number of clusters and the clustering algorithm. A more unbiased data driven feature selection can be used to overcome both the sparse sampling problem and multiple testing issue.

Singular Value Decomposition (SVD) and Independent Component Analysis (ICA) are popular dimension reduction techniques with different operating characteristics. Briefly, SVD is a factorization method that decomposes the data into a set of mutually orthogonal "eigentraits" that are sorted according to variance explained [9,10]. ICA decomposes the expression data into a set of statistically independent modes that we term as "ICAtraits". The statistical independence between modes is estimated by optimizing a contrast function, such as kurtosis or mutual information [11]. Unlike SVD, ICA components might differ based on the contrast function and number of underlying sources, which under a generative model is responsible for the variation in the data. We will refer to eigentraits and ICAtraits as "meta-traits", both of which are built from the linear combinations of the original set of expression traits. By analyzing only the most relevant meta-traits it is possible to capture major biological trends while potentially averaging out the gene-specific noise [10]. We used both ICA and SVD approaches as they capture different sources of variation. The more widely used method of SVD makes the implicit assumption of underlying gaussian sources when maximizing the variance explained by the uncorrelated features. If this assumption is not valid, then the orthogonal dimensions may be combinations of two or more distinct biological signals [12]. ICA is more sensitive to sources that exert independent influence on the data and is ideal for detecting mixtures of higher order statistics. It has been applied, to a wide variety of problems such as face recognition [13], image analysis algorithms [14], and for pathway enrichment in breast cancer data [15].

Both SVD and ICA have been previously applied to gene expression data [10,16-19]. SVD based methods like Principal Components Analysis (PCA) have found applications in studying oscillation profiles from genome-wide expression measurements [20,21], inferring network connectivity from expression data [22], and tissue sample classification [23,24] while ICA has been used for pattern recognition in tumor microarray data [19,25,26], to discover functional modules in microarrays [18] and in other more general signal processing applications [11]. PCA has also been explored in the context of QTL mapping. However, these mapping methods have been applied to small subsets of genes or a limited number of quantitative traits [27-30], but not to eQTL data.

In this study, SVD and ICA were applied to a well studied eQTL data set in yeast. The resulting meta-traits, which are mutually uncorrelated, can be thought to represent independent trends in expression variation [10]. We used the top 20 ranked SVD and ICA meta-traits to map eQTLs. We identified 21 eQTLs, 11 of which have not been previously described. Finally, we discuss the performance of both SVD and ICA with respect to capturing patterns in expression variation and linkage mapping.

## Results and Discussion

### Single Trait Analysis

We used data previously described by Brem et al [31] who measured gene expression levels of 6216 ORFs in 112 segregants derived from a cross between the *Saccharomyces cerevisiae *strains BY and RM. The expression level of each gene was treated as a quantitative trait (which we will refer to as gene expression trait) and eQTL were identified by linkage analysis using 3312 genetic markers distributed across the genome.

**Figure 1:**
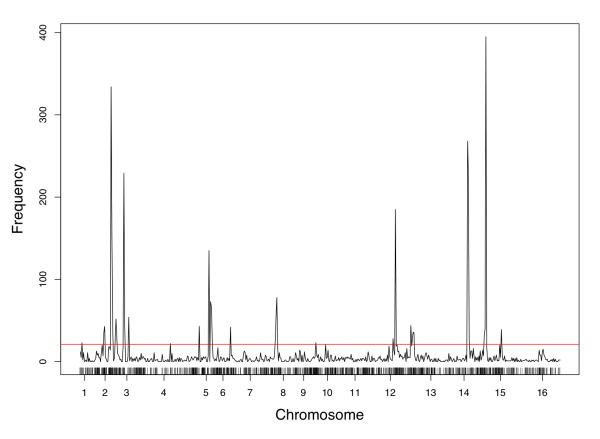
**Genome-wide distribution of linkages from single trait analyses**. The yeast genome was divided into non-overlapping 20 kb bins and the number of significant linkages to that bin was recorded. In total, 5013 gene expression traits showed significant linkage (FDR = 0.05) in the single trait analyses. The probability of any bin having 20 linkages or more by chance is less than 2.1E-4. This is denoted by the solid red line (Methods). Details about each linkage hotspot are summarized in Table 1.

Our goal was to investigate the use of data reduction techniques for mapping eQTL and to compare it with traditional single trait analyses. To this end, we first performed a genome-wide linkage analysis on each of the 6216 gene expression traits by standard regression techniques [32]. Each trait was tested for linkage at all 3312 markers, which amounts to approximately 8 million hypothesis tests. Significant linkages were detected for 5013 traits at a false discovery rate (FDR) of 0.05 (see Methods) [33]. Loci with widespread genetic effects were identified by dividing the genome into non-overlapping 20 kb bins and counting the number of linkages in each interval (Figure 1). Sixty percent of all linkages fell in 36 bins that had more than 20 linkages. With more segregants compared to a previous study [8], we identify a larger set of gene expression traits that link to each of the previously described eQTL hotspots (see Additional file 1). The linkage hotspots derived from single trait analyses are summarized in Table 1, and provide the necessary baseline to compare the eQTL analyses based on meta-traits too.

**Table 1:** Summary of single trait linkage analyses

**Group**	**Bin: Chromosome, Coordinate**	**N**	**X/T**	**P-adj**	**Common GO Annotation**
1	I: 52357	23	10/215	< 0.001	lipid/fatty acid metabolism
2	II: 368991	74	NA	NA	NA
3	II: 554641	514	80/213	< 0.001	ribosome biogenesis
4	II: 667104	99	22/213	< 0.001	cytoplasm organization and ribosome bio-genesis, rRNA processing
5	III: 90986	265	49/99	< 0.001	amino acid biosynthesis
6	III: 201166	53	5/8	< 0.001	regulation of transcription, mating-type specific
7	V: 116389	40	3/13	< 0.001	pyrimidine base biosynthesis
8	V: 422588	287	44/171	< 0.001	cytosolic ribosome
9	VII: 55461	42	10/252	0.001	cytoplasm organization and ribosome bio-genesis
10	VIII: 111679	153	18/100	< 0.001	conjugation, response to pheromone
11	X: 329085	21	NA	NA	NA
12	XII: 671271	182	24/45	< 0.001	ergosterol/sterol metabolism
13	XII: 1051813	44	15/84	< 0.001	helicase activity, telomerase maintenance
14	XIII: 49969	65	3/5	0.008	serine family amino acid metabolism
15	XIV: 486861	511	61/83	< 0.001	mitochondrial ribosome
16	XV: 172654	458	37/218	< 0.001	carbohydrate metabolism
17	XV: 563943	38	18/46	< 0.001	oxidative phosphorylation/respiratory- chain phosphorylation

eQTL positions of the 5013 traits for which linkage was detected at an FDR cutoff of 0.05, were binned into 20 kb bins and the chromosomal coordinates of each linkage hotspots that have more eQTLs than expected by chance, the number of traits linking to the hotspot (N), the number of those traits that have the over-represented GO attribute (X), the total number of traits that have this attribute (T) and over-represented GO annotation terms are reported.

### Dimension reduction using SVD and ICA

**Figure 2:**
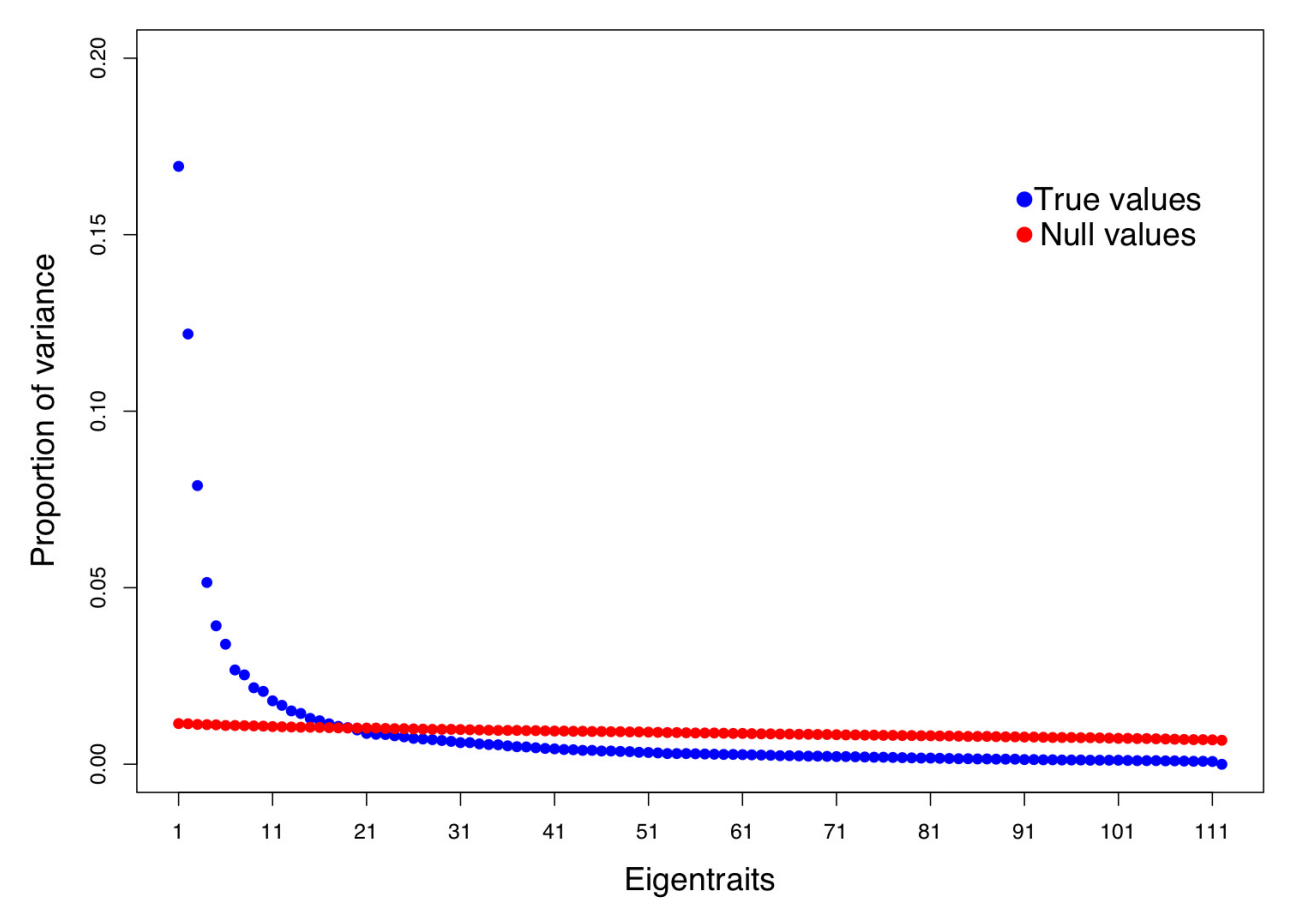
**Proportion of Variance explained by each eigentrait**. Singular value decomposition results in dimensions that we refer to as "eigentraits", which are ranked according to how much variation in the dataset they explain. The distribution of variance explained for the observed and null data are shown as blue and red circles, respectively.

SVD analysis was performed to reduce the dimensionality of the data from the original 6216 expression traits to 112 eigentraits, where each eigentrait is a linear combination of all gene expression traits. The proportion of variance explained for each eigentrait relative to total variation in the data set is shown in Figure 2. Eigentraits that explained more variation than expected by chance were identified by comparing with a null-dataset (see Methods), and in total, the top 20 eigentraits, which collectively account for approximately 72% of all variation, were selected for further study.

Independent modes from an ICA based decomposition were sorted by the Liebermeister criterion [34] (see Methods). To enable comparison between the trends in variation captured by the two different methods we selected the top 20 ICAtraits consistent with the 20 significant eigentraits that were analyzed.

As each meta-trait is a linear combination of all 6216 gene expression traits, it would be informative to infer the set of specific genes that make the largest contribution to each one. To identify these genes, we calculated the correlation between each of the 6216 gene expression traits with each meta-trait and determined significantly correlated genes by permutations (see Methods). As shown in Tables 2 and 3, the number of significantly correlated (*p *< 0.0001) gene expression traits range from 5 to 1919 (see Additional file 2) and 3 to 1448 (see Additional file 3) for each eigentrait and ICAtrait, respectively.

**Table 2:** Summary of Eigentrait linkage and Gene Ontology analyses

**Trait**	**QTL**	**N**	**X/T**	**P-adj**	**GO over-representation**
1	II:554641	1919	190/252	< 0.001	ribosome biogensis, rRNA processing
2	III: 90986, XV: 172654	601	92/180	< 0.001	amino acid metabolism, carbohydrate metabolism, ligase activity
3	III:201166	196	20/94	< 0.001	Ty element transposition
			4/4	< 0.001	mating pheromone activity
4	II:530481, XII:473036	13	4/5	< 0.001	asparagine catabolism, cellular response to nitrogen starvation
5	II: 554641, III: 201166	38	3/8	< 0.001	SRP-dependent cotranslational protein-membrane targeting
6	II:310928, XIV:486861	366	227/1017	< 0.001	Cellular protein/macromolecule metabolism
7	II:554641, V:116389, VIII:111686	37	6/100	0.007	conjugation, sexual reproduction, response to pheromone
8	XII: 796771, XII: 1056097	47	15/84	< 0.001	helicase activity, telomerase maintenance, mitotic recombination
9	0	74	8/106	< 0.007	Bud neck
10	XII: 660992	41	9/25	< 0.001	ergosterol/sterol/steroid/lipid metabolism/biosynthesis
11	0	8	NA	NA	Unknown
12	V:116389, XIII:379975	15	NA	NA	Unknown
13	0	8	NA	NA	Unknown
14	V:116389	8	2/13	0.039	pyrimidine base biosynthesis
15	IV:435872	5	NA	NA	Unknown
16	0	0	NA	NA	NA
17	0	0	NA	NA	NA
18	0	6	NA	NA	Unknown
19	IV:518397	5	3/3	< 0.001	sodium ion transport/sodium transport, ATPase activity
20	XV:538788	5	NA	NA	Unknown

For each eigentrait, we report the number and position of any significant linkages detected, the number of traits that are significantly correlated with a specific eigentrait (N), the number of correlated traits that have the over-represented GO attribute (X), the total number of traits that have this attribute (T) and also the over-represented GO annotation term. Two of the twenty have no correlated traits that can be called significant while six showed no significant over-representation of any biological function and remaining twelve show over-representation of certain biological functions that are consistent with previously reported trends of large scale expression variation like the mating locus, auxotrophic markers and stress response among others. Novel eQTLs detected for eigentraits 4 and 19 are described in more detail in the text.

**Table 3:** Summary of ICAtrait linkage and Gene Ontology analyses

**Trait**	**QTL**	**N**	**X/T**	**P-adj**	**GO over-representation**
1	XII: 423789, XV: 179409	189	58/1017	< 0.001	mitochondrion
2	III: 201166	45	5/8	< 0.001	mating-type specific/mating-type specific transcriptional control
3	0	77	26/108	< 0.001	nitrogen compound biosynthesis, amine biosynthesis
4	XII: 473036	12	4/5	< 0.001	asparagine catabolism
5	0	3	NA	NA	NA
6	II: 554641	1448	54/83	< 0.001	mitochondrial ribosome
7	V: 422588	63	26/94	< 0.001	retrotransposon nucleocapsid/VLP/Virus-like particle
8	III: 90986	49	8/13	< 0.001	nitrogen compound biosynthesis, amine biosynthesis
9	X: 329085	22	4/6	< 0.001	alcohol dehydrogenase activity/alcohol dehydrogenase (NAD) activity, fermentation
10	XIV: 449639	304	225/1017	< 0.001	mitochondrion, mitochondrial ribosome
11	XII: 660992	83	22/37	< 0.001	sterol metabolism, sterol biosynthesis
12	V: 116389	9	3/13	< 0.001	pyrimidine base biosynthesis
13	XV: 563943	184	125/1017	< 0.001	oxidative phosphorylation/respiratory- chain phosphorylation, mitochondrion
14	VIII: 111686	42	16/100	< 0.001	conjugation, sexual reproduction
15	IX: 141014 XIII: 395391	8	NA	NA	NA
16	IV: 1501558, XI: 298361,	47	15/84	< 0.001	helicase activity, telomerase-independent
	XII: 824230, XII: 1056097				telomere maintenance
17	IV: 527484	6	3/3	< 0.001	sodium ion transport/sodium transport/sodium:solute transport
18	XII: 423789, XV: 179409	32	2/2	0.015	NA
19	XIII: 40447	14	4/5	< 0.001	acid phosphatase activity
20	0	63	NA	NA	NA

For each ICAtrait, we report the number and position of any significant linkages detected, the number of traits that are significantly correlated with a specific ICAtrait (N), the number of correlated traits that have the over-represented GO attribute (X), the total number of traits that have this attribute (T) and also the over-represented GO annotation term. Three of the 20 show no over-representation of biological functions while for remaining 16 both previously described and novel GO categories with associated eQTL were detected. Analysis of ICAtrait 7 and 9 are described in more detail in the text.

To identify general biological themes, we performed a Gene Ontology (GO) analysis for each set of significantly correlated expression traits across the top 20 meta-traits (Table 2 and 3, [35]). For 12 of the 20 eigentraits, the significantly correlated genes show an overrepresentation of GO terms related to specific biological processes such as budding, amino acid, sterol and carbohydrate metabolism, and ribosome biogenesis, which is generally consistent with previous analyses [8]. Similar enrichment of GO terms were found for 17 out of 20 ICAtraits, with additional categories such as retro-transposon, alcohol dehydrogenase and acid phosphatase activity being detected (Table 3). In addition to the enriched set of traits with common biological process that have been described in earlier eQTL studies, we identified four novel group of traits that have not been identified through linkage analysis to date. Two of them, Eigentraits 4 and 19, are defined by clusters of genes with similar function and will be described in more detail below. The other two, ICAtraits 7 and 9, are associated with retro-transposon activity and alcohol dehydrogenase activity, respectively.

### Linkage Analysis of Meta-traits

**Figure 3:**
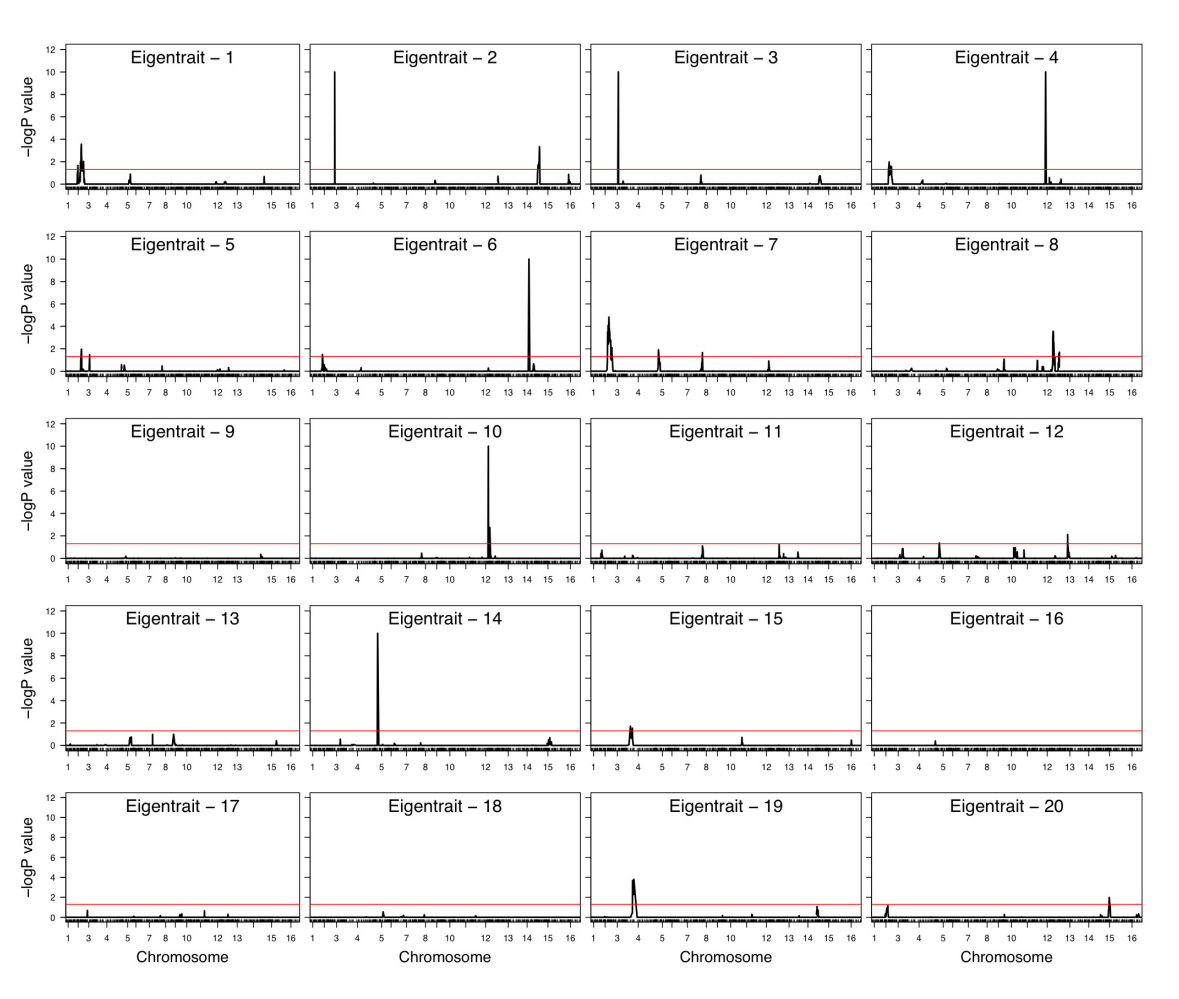
**Genome-wide linkage analysis for the top 20 eigentraits**. In each linkage profile, the negative log p-value of the linkage statistic for each eigentrait is plotted against the genomic position of all the markers. Significance is determined by a GWER < 0.05. Fourteen of the 20 eigentraits show linkage to at least one QTL. Tolerance is set at 1E-10 for p-values equal to zero.

**Figure 4:**
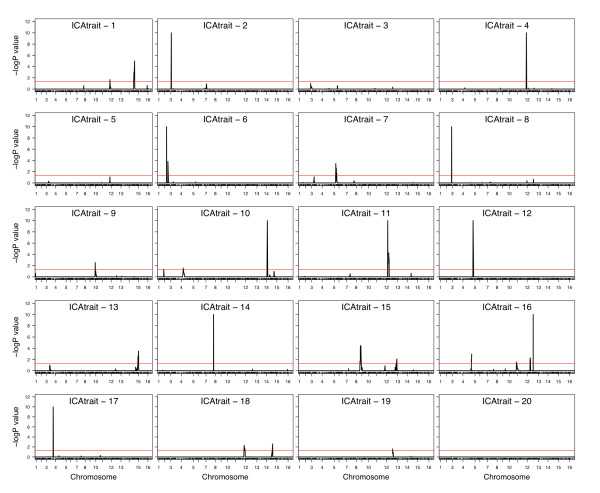
**Genome-wide linkage analysis for the top 20 ICAtraits**. In each linkage profile, the negative log p-value of the linkage statistic for each ICAtrait is plotted against the genomic position of all the markers. Significance is determined by a GWER < 0.05. Seventeen of the 20 ICAtraits show linkage to at least one QTL. Tolerance is set at 1E-10 for p-values equal to zero.

For the top 20 meta-traits, we performed a genome-wide linkage analysis using 3312 genetic markers that were genotyped in each segregrant. Linkage analysis was performed by regressing marker genotypes on trait values for each meta-trait and significance was determined by permutations. We considered markers to be significant according to a permutation based genome-wide error rate of 5% as significant [36]. The genome-wide linkage analyses for each eigentrait and ICAtrait is shown in Figure 3 and Figure 4, respectively. In total, 14 eigentraits demonstrate significant linkage to one or more places in the genome resulting in a total of 15 unique eQTL. Eigentraits 2, 4, 5, 6, 8 and 12 each link to 2 eQTL while eigentrait 7 links to three eQTLs. Similar analysis of the ICAtraits resulted in 20 unique eQTLs being detected (*p *< 0.05, Table 3) that were distributed over 17 ICAtraits. ICAtraits 1, 15 and 18 showed linkages to two eQTLs while ICAtrait 16 linked to four eQTLs. At a genome-wide error rate of 5 %, we expect two false positive among the total of 45 eQTL deemed significant.

Ten of 21 unique eQTLs identified map to previously described regions of *trans*-regulatory hotspots [1] that regulate groups of genes with shared biological functions such as amino acid catabolism (eigentrait 2, ICAtrait 8), mating (eigentrait 3, ICAtrait 2), mitochondria (eigentrait 6, ICAtrait 1), and heme/fatty acid metabolism (eigentrait 10, ICAtrait 11). For each of these meta-trait, we observed a corresponding enrichment of related GO terms in the set of correlated traits (Table 2 and Table 3).

**Figure 5:**
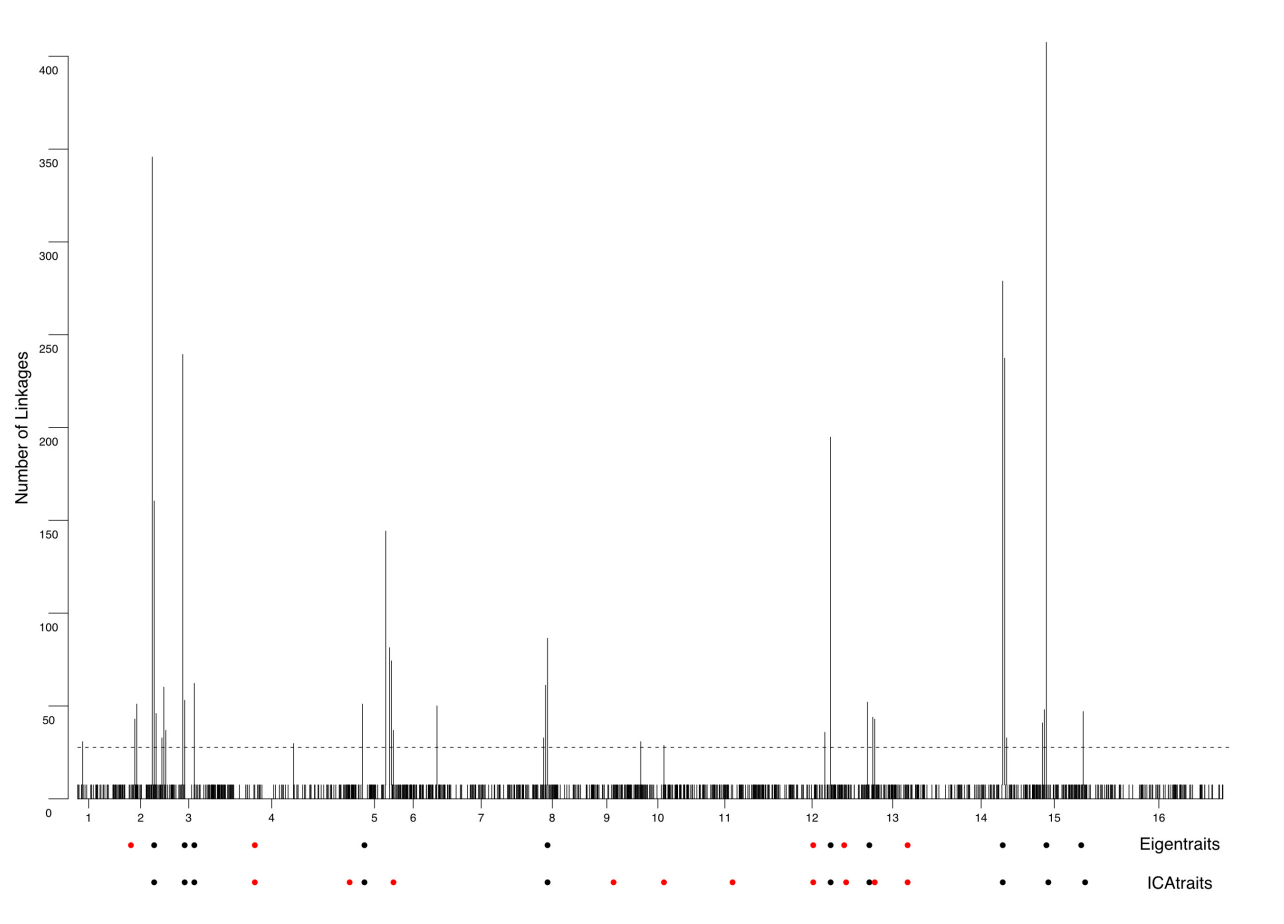
**Overlap between results from single trait linkage scans with linkage analysis of eigentraits**. The y-axis of the top panel represents the number of linkages that fall in each 20 kb non-overlapping regions spread across the genome while along the x-axis the position of genotyped markers at which linkage was estimated is marked. The next two rows of solid circles mark the position of eQTLs detected for eigentraits and ICAtraits, respectively. The position is aligned with the markers on the x-axis of the top plot. The red solid circles represents novel eQTLs while black represents previously described eQTL.

The genome-wide linkage results of meta-traits derived from SVD and ICA show considerable overlap (Figure 5). Overlapping eQTLs primarily correspond to loci that exert widespread expression variation and include eQTLs with strong *trans*-acting effects, which is consistent with the fact that both SVD and ICA were able to capture the major sources of variation by its top ranked components. Of the eleven new eQTL, four showed evidence for *cis*-linkage, which will be discussed in more detail below. The other linkages map to regions in the genome that either show no significant enrichment of linkages from the single trait analysis or there is no obvious gene to explain the enrichment of GO annotation for that meta-trait.

**Figure 6:**
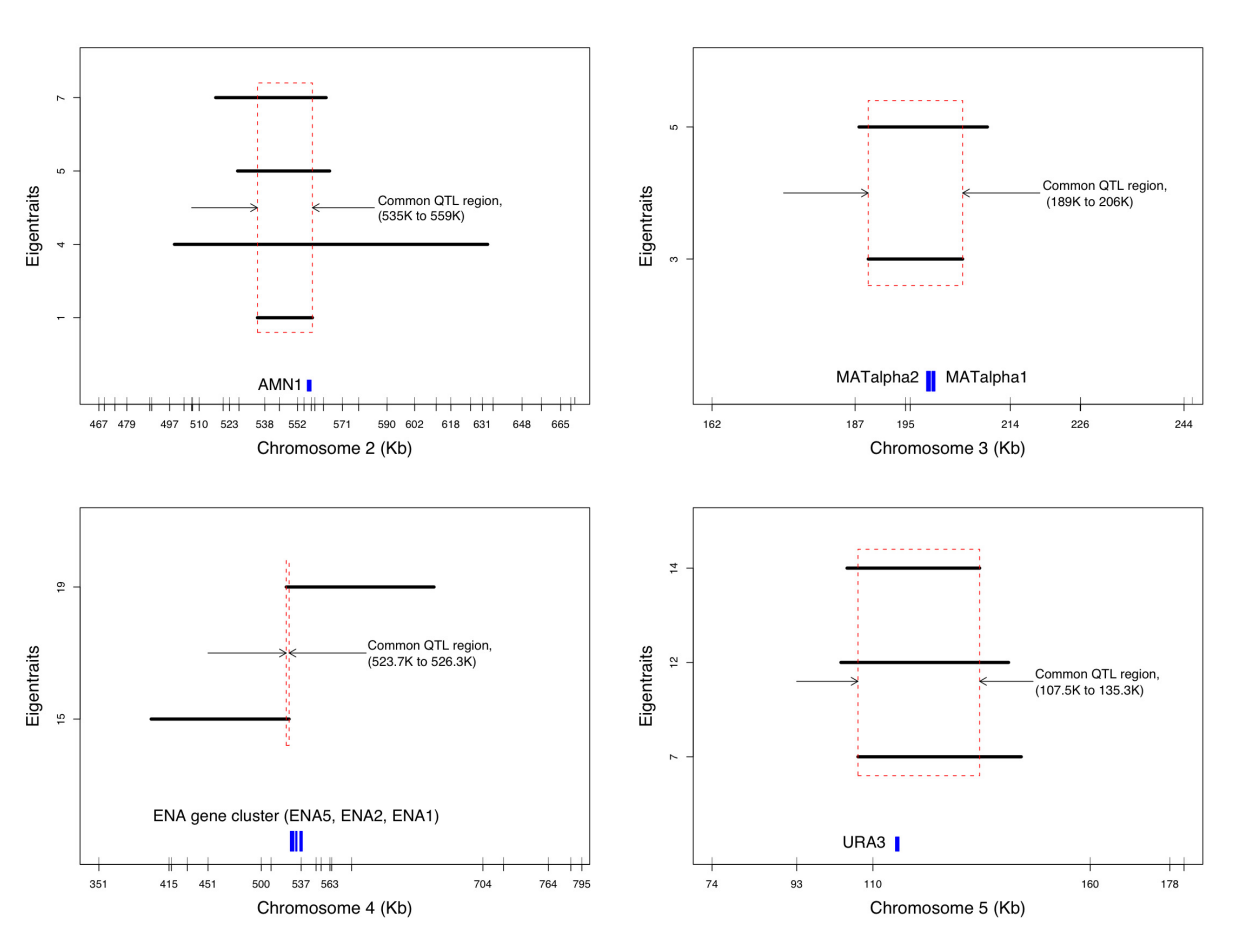
**Overlap of QTL location across orthogonal eigentraits**. In each plot the length of overlap between eQTL locations across multiple eigentraits is represented by the 1 LOD support interval that are denoted by black bars. Also, for each plot the location of the putative regulator, which might explain the enrichment of certain GO functional categories for traits correlated with specific eigentraits is represented along the x-axis by blue vertical bars.

Figure 6 summarizes shared linkages between eigentraits. Each plot corresponds to a common eQTL that shows linkage to different eigentraits. These regions could potentially harbor either pleiotropic eQTL or two or more linked eQTLs.

### Analysis of Putative cis-Acting Meta-trait Linkages

In traditional linkage scans where traits are analyzed one at a time, a *cis*-linkage is characterized as an expression trait showing linkage to its own genomic location. In the setting of meta-trait linkage scans, we define a *cis*-linkage of a meta-trait as linkage to the genomic position of a trait that is also significantly correlated with this meta-trait. In the following sections we describe in depth analyses of four novel *cis*-linkages that were not described in previous eQTL studies. These include eigentraits 4 and 19, and ICAtraits 7 and 9, which show significant enrichment of GO annotation terms.

#### Cis-regulation of Asparaginase Metabolism

Eigentrait 4 has a strong *cis*-linkage on chromosome 12 (Figure 3). The region harbors genes involved in the catabolism of asparaginase during nitrogen starvation (*ASP3*-1, *ASP3*-2, *ASP3*-3, *ASP3*-4; see Additional file 4). There are 13 expression traits that are significantly correlated with this eigentrait (*p *< 0.0001) and the top four correlated genes make up the tandem array of asparaginase (*ASP*) genes. The nine remaining significantly correlated genes are strong candidates for participating in the asparaginase metabolism network. Comparative sequence analysis with the published draft RM genome sequence [37] revealed the absence of the *ASP *gene cluster in the RM strain, which is consistent with the eQTL being supported by the highest F-statistic among all linkages and the small number of genes that are principal contributors to this particular eigentrait.

#### Cis-regulation of Sodium Transport

Eigentrait 19 also shows evidence of *cis*-linkage on chromosome 4. This eigentrait is enriched for genes involved in sodium transport. Genes with the highest correlation to eigentrait 19 are the group of *ENA *genes *ENA5*, *ENA2 *and *ENA1*, which are members of the sodium efflux ATPase family and span the chromosome 4 region that surrounds the linked eQTL (see Additional file 4). Sequence analysis of the RM draft genome for the *ENA *group of genes shows alignment hits to a single copy of the *ENA *gene on supercontig 1, which suggests that copies of the *ENA *genes have been deleted along the RM lineage as one would expect an alignment to all three copies otherwise. This observation is consistent with the hypothesis of possible copy number changes existing at this locus between the two strains. However, the draft status of the genome precludes a more definite inference of copy number change.

#### Cis-regulation of Retrotransposon Activity

Sixty-three genes are significantly correlated with ICAtrait 7 (*p *< 0.0001) and one significant eQTL was mapped on chromosome 5 (Figure 4). As the GO analysis of the 63 genes shows, a large proportion of these genes are transposable elements and/or involved in the process of retro-transposition. The multipoint linkage profile of the eQTL on chromosome 5 shows the location of a subset of genes that lie in the 1 LOD support interval (see Additional file 5). The gene YERCTy1-1, a retrotransposon of the Ty1 family, is present in the eQTL support interval and also is significantly correlated with ICAtrait 7. This supports the hypothesis that YERCTy1-1 is a possible candidate gene underlying this eQTL.

Further analysis of the sequence data was performed using the published RM draft sequence. The alignment of the gene as well as part of its upstream and downstream genomic sequence in the two strains points to a possible insertion-deletion polymorphism spanning the whole retro-transposon. A caveat for this observation is a possible alignment error, which might result due to the high degree of homology within different transposable element families and the unassembled state of the RM genome. This does not rule out the presence of a polymorphism underlying the eQTL, but makes detection using currently available sequence data more difficult.

#### Cis-regulation of Alcohol Dehydrogenase Activity

ICAtrait 9 is comprised of 22 significantly correlated genes that are enriched for alcohol dehydrogenase activity and fermentation. Of the 22 genes, only YJL056C falls in the 1 LOD support interval of the eQTL on chromosome 10 (see Additional file 5). YJL056C is a transcription factor that binds to zinc-responsive promoter elements to induce transcription in the presence of zinc. The other genes in the correlated set include the *ADH *group of enzymes that are alcohol dehydrogenases involved in fermentation and the glycolytic pathway. The transcription of these genes is activated in times of zinc deficiency, which is also supported by the presence of zinc transporters like *ZRT3 *and *ZRT2 *in the set of correlated genes. These observations suggest that *ZAP1 *is a possible candidate gene underlying the eQTL. We performed multiple sequence alignment of the *ZAP1 *coding sequence from the two *Saccharomyces cerevisiae *strains, BY and RM as well as two related species *Saccharomyces mikatae *and *Saccharomyces paradoxus*. Of the 19 single nucleotide polymorphisms that were found, 10 resulted in a non-synonymous change (see Additional file 6). These polymorphisms are strong candidates for further computational and functional analysis in order to narrow down the eQTL underlying ICAtrait 9 to the nucleotide level.

## Conclusion

Analyzing low dimensional representations of high-dimensional data through techniques such as SVD and ICA is a useful approach for studying the genetic architecture of gene expression variation [1]. We find that the meta-trait linkage analysis approach is complementary to traditional single trait linkage scans, which are inefficient in exploiting the complex correlation structure that exists among gene expression levels. In addition, this approach results in a smaller number of traits to analyze, thus increasing statistical power by attenuating the multiple testing problem.

Randomizing parental yeast genomes through genetic crosses induces widespread changes in expression, which allows the contribution of genetic variation to gene expression changes, to be systematically probed and makes it an appealing situation to apply dimension reduction methods. By applying SVD and ICA to the unfiltered expression matrix, we are able to focus our analysis on the most biologically meaningful meta-traits that we term "eigentraits" and "ICAtraits", respectively. In this low dimensional snapshot, each meta-trait is uncorrelated with the others and is a weighted average of all the 6216 traits and hence can be analyzed independently of others. The approach we outline for identifying significantly correlated genes for each meta-trait allows gene sets to be identified and subjected to further bioinformatics and functional analyses.

The complementary nature of this study compared to single trait analyses is supported by the fact that eigentraits with some of the highest singular values map to previously described strong effect eQTLs such as *LEU2*, *URA3*, *MAT *locus, Msn2/4 targets, and *AMN1*. These results are also reinforced by the ICAtrait analysis that uncovered similar large effect QTLs. Furthermore, the utility of both approaches is demonstrated by the detection of eleven novel eQTLs that supplements our understanding of the genetic architecture of gene expression differences between these two *S. cerevisiae *strains. These include four novel *cis*-linkages that were studied in greater detail. Two of them map to tandem arrays of genes with similar functions that are involved in asparaginase metabolism and sodium ion transport. Comparative sequence analysis of the BY and RM strains sugests that these gene clusters have been lost in RM and is consistent with reports of copy number changes at these loci in non-laboratory yeast strains [38]. These two meta-traits also show differential expression between the two parental strains at the marker with the highest linkage statistic (see Additional file 7). This is interesting as the two strains have evolved in very different ecological niches and might depend on different nutrient sources for survival. Using ICAtraits, two additional *cis*-linkages were identified and found to be associated with differences in retro-transposititon and alcohol dehydrogenase activity. Analysis of the linkage region at a finer scale also provided strong candidate genes that might be potential regulators of these expression differences.

Despite the strict genome-wide threshold that we used, there were four cases of eQTLs common between eigentraits (Figure 6). Such observations coupled with the orthogonal property of meta-traits is consistent with either pleiotropy or coordinate linkage between two closely spaced eQTLs. However, it is important to note that such inferences are tenuous because a single biological signal may be captured by multiple eigentraits. This cannot be ruled out as the eigentraits, being linear combinations of all expression traits, are hard to interpret qualitatively. Interestingly, there is only one case of linkages being shared between ICAtraits, suggesting that ICA is better at discriminating between the different biological signals present in the data. Furthermore, ICA identified a larger set of novel eQTLs compared to SVD. This may be due to ICA's estimation of statistically independent components in higher order moments that detects non-normally distributed trends, while SVD relies on the absence of correlation in second order moments of normal trends. The non-normally distributed or long-tailed distribution in this dataset is expected based on the finding from single trait analyses that there exists a small number of linkage "hotspots" that are responsible for most of the variation in the dataset.

Another scenario where the interpretation of the results might be potentially misleading is when the meta-traits capture technical artifacts in the microarray experiment, for example signal that is driven by cross-hybridization instead of true differential expression. One approach to assess the effect of cross-hybridization on the eQTL data is to test the hypothesis that paralogous genes are enriched among significantly correlated meta-traits. For example, eigentrait 8 consists of 47 significantly correlated traits, of which 7 are paralogs (*YRF1-1*, *YRF1-2*, *YRF1-3*, *YRF1-4*, *YRF1-5*, *YRF1-6*, *YRF1-7*). Thus cross-hybridization among these genes may be influencing this eigentrait. Note, linkage analysis of eigentrait 8 identified two closely linked *cis*-eQTL on chromosome 12 (Figure 3). One of these *cis*-linked regions contains *YRF1-4 *and *YRF1-5*. Thus cross-hybridization of *YRF1-4 *and *YRF1-5 *with the other *YRF1 *paralogs could potentially explain this apparent *cis*-linkage. However, if this were true we would expect to see a *cis*-linkage at one of the other *YRF1 *genes, which we do not observe. Therefore, the linkages observed for eigentrait 8 appear to be robust, but the larger issue of technical artifacts due to cross-hybridization and other sources is important to keep in mind when interpreting eQTL studies.

In summary, we highlight the applicability of dimension reduction methods for studying large-scale patterns of variation in gene expression traits. We argue for the use of both SVD and ICA if there are no prior expectations about the different patterns of variation present in genome-wide expression trait measurements. It also represents an important tool in recovering previously undetected eQTL, for exploring the widespread but uncharacterized cases of pleiotropy, and provides the basis for a more detailed understanding about how regulatory variation manifests itself across transcriptional networks.

## Methods

### Data

We used data previously described by [31]. Briefly, 112 segregants from a cross between the yeast strains BY and RM were genotyped at 3312 markers using high density Affymetrix oligonucleotide arrays that were designed against the BY genome. The marker set covered more than 99% of the genome and of the whole marker set, 1226 genotypes are unique across the segregants and were retained for further analysis. Expression levels of 6216 ORFs measured using cDNA microarrays were normalized by spatial lowess smoothing and dye, array and sample effects were accounted for using a mixed-model ANOVA [39].

### Singular Value Decomposition

The expression matrix **X **of 6216 (N) traits in 112 (M) segregants was mean centered for each trait to remove the baseline level of expression to focus on levels of expression variation. SVD was performed on the centered data to result in **U**, a N × M "eigensegregant" matrix, **D **a M × M diagonal "eigenweight" and **V**^*T*^, the M × M "eigentraits" matrix:

**X **= **U D V**^*T*^

The proportion of variance explained by each eigentrait, *v*(*e*_*i*_) was calculated as:

$$v(e_{i})=\frac{e_{i}^{2}}{\sum_{j=1}^{M}e_{j}^{2}}$$

where *e*_*i *_denotes the eigenvalue of the *i*th eigentrait.

The Shannon entropy of the data was calculated as:

$$d=\frac{-1}{\log(M)}\sum_{i=1}^{M}v(e_{i})\log(v(e_{i}))$$

To determine the significance threshold for eigentraits, we used Horn's procedure [40]. A null dataset with same dimensions as the original dataset was constructed and those eigentraits with eigenvalues greater than those of their respective random components were considered for the analysis.

### Independent Component Analysis

The *fastICA *algorithm implemented in the *fastICA *R package [41,42] was used to extract 112 modes from the expression matrix **X**, where each trait was mean centered. Independent Component Analysis decomposed **X **into its constituent source matrix **S**, and mixing matrix **A**.

**X **= **S A**

The modes of **A**, which are linear combinations of the expression traits form the ICAtraits. The default Liebermaster contrast value from *mlica *R package [41,43] was used to sort the ICAtraits. The contrast is a measure that combines the data variance and also non-normality. The top 20 of the sorted ICAtraits were selected for further analysis to enable comparison with 20 significant eigentraits. Selecting the number of independent modes in an ICA analysis is by itself a current research topic. To enable comparison with the SVD analysis we selected the same number of 112 independent modes in the ICA decomposition of the data.

### Correlation Analysis

To identify genes that make a significant contribution to a meta-trait, we calculate the correlation of each of the 6216 gene expression traits to each of the 20 meta-traits. To estimate the significance threshold for the correlation measure, 1000 permutations of the segregants are performed and correlation with each eigentrait is estimated as described previously. The absolute value of the null correlations are pooled and threshold set at a p-value < 0.0001.

### Linkage Analysis

Genome-wide linkage analysis was initially performed on each expression trait separately. Briefly, for each trait we tested for linkage across all 3312 genetic markers using standard linear regression. To estimate null statistics, the gene expression of 112 segregants were permuted 10 times and linkage tested as before with all markers. The maximum statistic in each permutation was retained and used to calculate the p-value for each trait. The 5013 significant linkages at a FDR cut-off of 0.05, with an estimated upper bound of *π*_0 _= 0.152 of the expression traits showing no linkage [33], grouped together in 589 20 kb bins across each chromosome to identify regulatory hotspots. Adjacent bins around hotspots on chromosome 2, 3, 5, 8, 12 and 15 that had more significant linkages expected by chance were merged, to estimate the GO annotation overrepresentation associated with traits, as these likely represent the action of the same underlying QTL. Assuming that the distribution of significant linkages across the bins is random and follows a Poisson distribution, the probability that the number of linkages in a bin will be more than 20 is < 2.1e-4.

Similar single marker analysis was used to perform genome-wide linkage scans for each of the 20 meta-traits. The genome-wide error rate (GWER) of 0.05 was determined by estimating p-values from pooling together the maximal statistic across 3312 markers from 10,000 permutations of each meta-trait [36]. As there are only 20 traits, no correction of multiple hypothesis tests was performed while analysing meta-traits. As reported earlier in the text, the analysis of each set of 20 meta-traits result in one expected false positive linkage. Multipoint linkage was also estimated for those meta-traits for which novel eQTLs were described in more detail. These include eigentraits 4, 19 and ICAtraits 7, 9. The R/QTL package [44] was used to estimate the linkage score after computing genotype probabilities at 1 cM intervals.

## Authors' contributions

SB, JDS, and JMA conceived and designed the experiments. SB analyzed the data. SB and JMA wrote the manuscript with contributions from JDS.

## Supplementary Material

Additional file 1 **Linkage hotspot information**. List of all significant genes from the single trait linkage analyses where the linkage results were binned into 20 kilobase bins across each chromosome to identify "hotspots". Using a poisson distribution, the probability of having 20 linkages present in a bin by chance is < 2.1E-4. 17 such "hotspots" were identified. Click here for file

Additional file 2 **Eigentrait composition**. List of all transcripts that are significantly correlated with each of the top 20 eigentraits. Click here for file

Additional file 3 **ICAtrait composition**. List of all genes that are significantly correlated with each of the top 20 ICAtraits. Click here for file

Additional file 4 **Multipoint linkage profile of Eigentrait – 4 and Eigentrait – 19**. The multipoint linkage profile of Eigentrait 4 and 19 are plotted in the upper and lower half of the figure, respectively. In each case, only a section of the chromosome spanning the maximum LOD score is plotted with the 1 LOD support interval denoted by solid black bar. For Eigentrait 4, the position of the tandem array of three genes that are involved in asparaginase catabolism are represented by blue vertical bars. Similarly, for Eigentrait 19 the position of the tandem array of sodium ion efflux genes are denoted by blue vertical bars. Click here for file

Additional file 5 **Multipoint linkage profile of ICAtrait – 7 and ICAtrait – 9**. Similar to S4, the multipoint linkage profile spanning the maximum LOD score for ICAtrait 7 and 9 are plotted in the upper and lower half of the figure, respectively. In both plots, the position of a subset of genes that lie in the 1 LOD support interval represented by solid black bar is shown. For ICAtrait 7, only YERCTy1-1, a retrotransposon, shows significantly correlation while for ICAtrait 9, only *ZAP1 *shows a significant correlation with the ICAtrait. Click here for file

Additional file 6 **Multiple sequence alignment of YJL056C**. CLUSTALW was used to create a sequence alignment of the protein encoded by YJL056C/*ZAP1 *from two strains of *Saccharomyces cerevisiae *and two related species *Saccharomyces mikatae *and *Saccharomyces paradoxus*. The alignment output was then run through BOXSHADE to generate a colored output based on the conservation and degree of identity of the aligned residues. Nineteen SNPs were detected in the protein alignment, of which 10 were non-synonymous. Click here for file

Additional file 7 **Differential expression of two Eigentraits showing cis-linkage**. The top panel represents Eigentrait 4 and the bottom panel represents Eigentrait 19. Scatter plot of eigentrait values on the y-axis against the parental genotypes at the marker with the highest linkage statistics on the x-axis shows marked differential expression in the segregants (*p *< 0.0001). Click here for file
