# Supplementary figures and images for: Mapping gene expression quantitative trait loci by singular value decomposition and independent component analysis
Source: BMC Bioinformatics. 2008 May 20;9:244. doi: 10.1186/1471-2105-9-244 (PMC2424053; doi:10.1186/1471-2105-9-244)

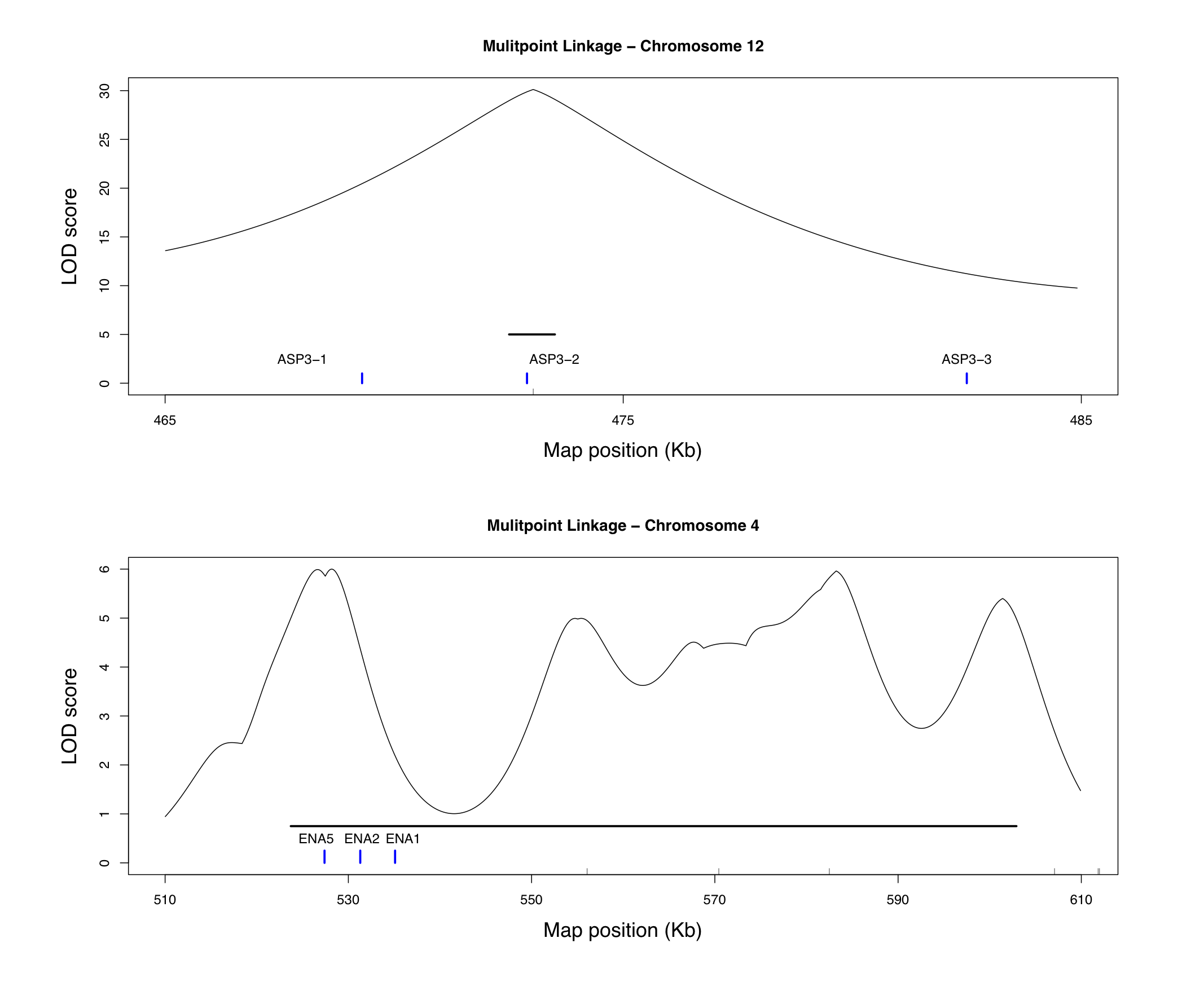

Supplement: Additional file 4 — Multipoint linkage profile of Eigentrait – 4 and Eigentrait – 19. The multipoint linkage profile of Eigentrait 4 and 19 are plotted in the upper and lower half of the figure, respectively. In each case, only a section of the chromosome spanning the maximum LOD score is plotted with the 1 LOD support interval denoted by solid black bar. For Eigentrait 4, the position of the tandem array of three genes that are involved in asparaginase catabolism are represented by blue vertical bars. Similarly, for Eigentrait 19 the position of the tandem array of sodium ion efflux genes are denoted by blue vertical bars. [file 1471-2105-9-244-S4.tiff]

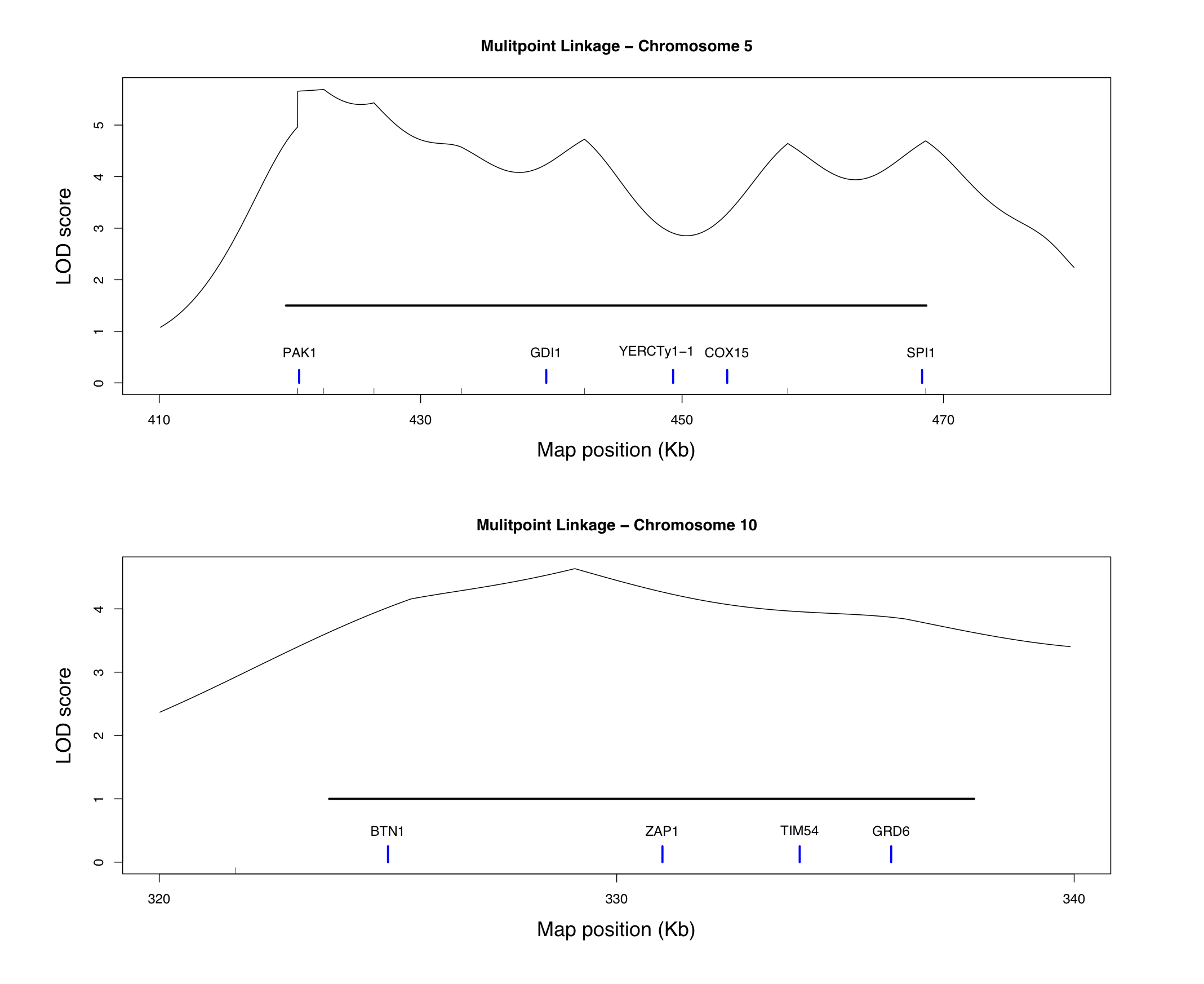

Supplement: Additional file 5 — Multipoint linkage profile of ICAtrait – 7 and ICAtrait – 9. Similar to S4, the multipoint linkage profile spanning the maximum LOD score for ICAtrait 7 and 9 are plotted in the upper and lower half of the figure, respectively. In both plots, the position of a subset of genes that lie in the 1 LOD support interval represented by solid black bar is shown. For ICAtrait 7, only YERCTy1-1, a retrotransposon, shows significantly correlation while for ICAtrait 9, only ZAP1 shows a significant correlation with the ICAtrait. [file 1471-2105-9-244-S5.tiff]

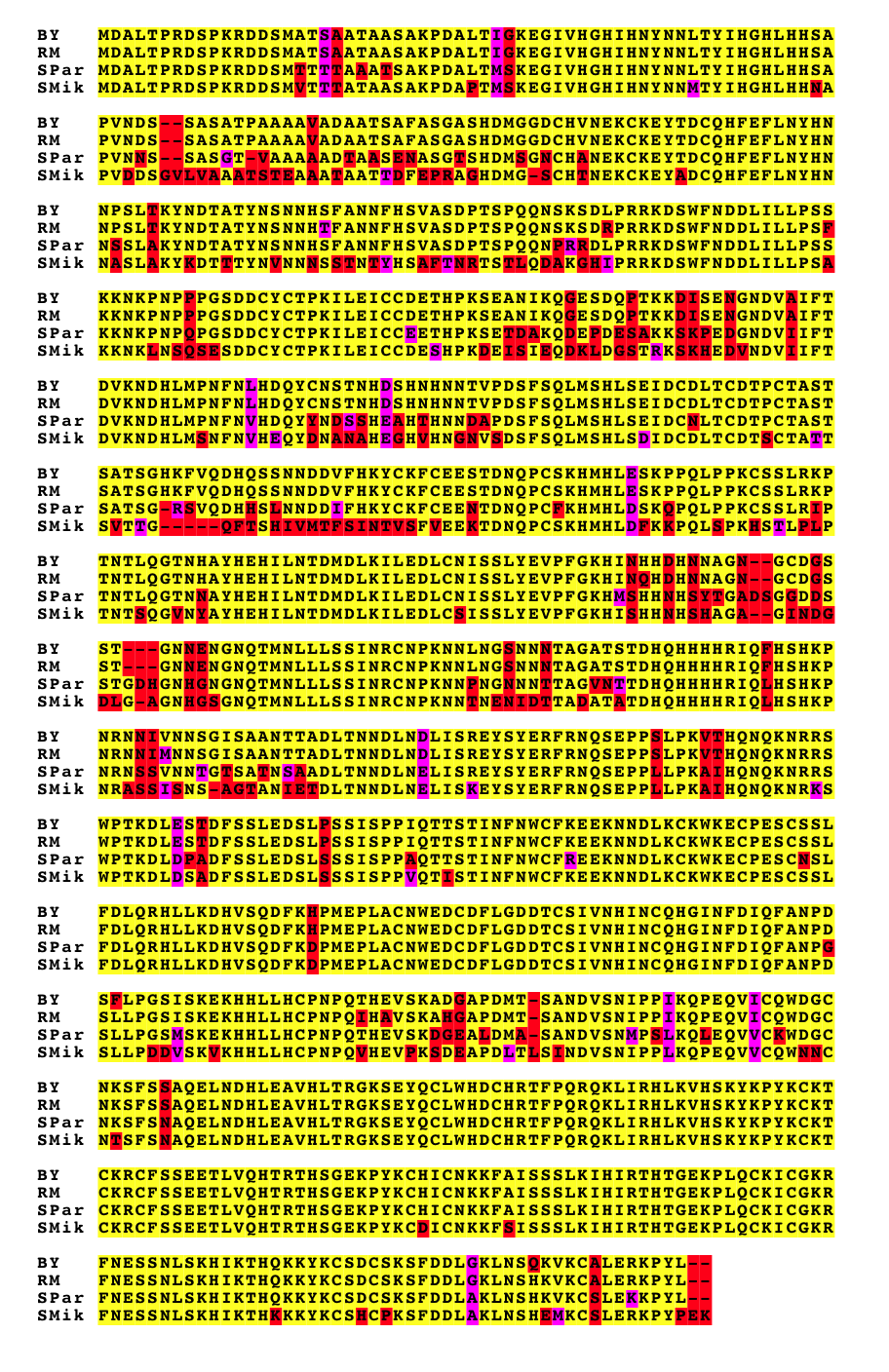

Supplement: Additional file 6 — Multiple sequence alignment of YJL056C. CLUSTALW was used to create a sequence alignment of the protein encoded by YJL056C/ZAP1 from two strains of Saccharomyces cerevisiae and two related species Saccharomyces mikatae and Saccharomyces paradoxus. The alignment output was then run through BOXSHADE to generate a colored output based on the conservation and degree of identity of the aligned residues. Nineteen SNPs were detected in the protein alignment, of which 10 were non-synonymous. [file 1471-2105-9-244-S6.tiff]

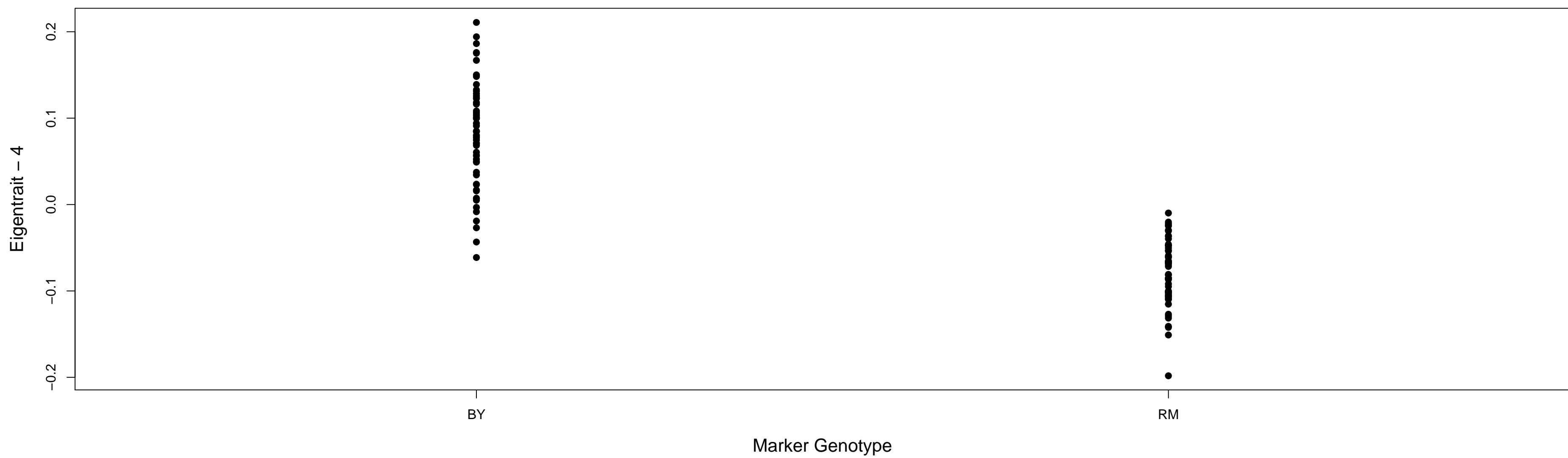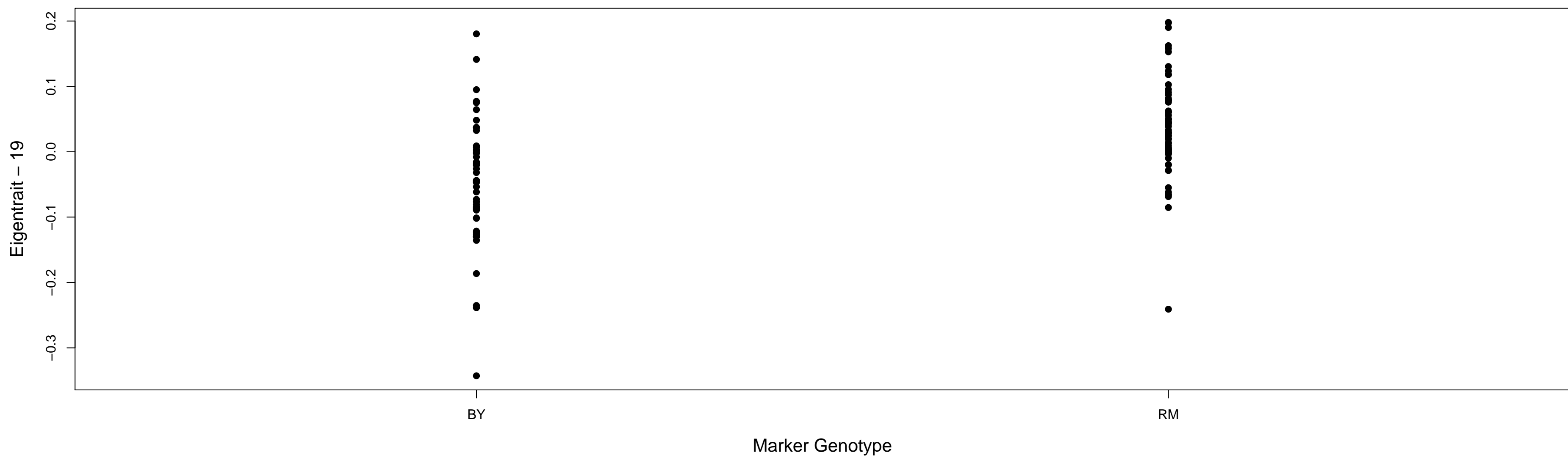

Supplement: Additional file 7 — Differential expression of two Eigentraits showing cis-linkage. The top panel represents Eigentrait 4 and the bottom panel represents Eigentrait 19. Scatter plot of eigentrait values on the y-axis against the parental genotypes at the marker with the highest linkage statistics on the x-axis shows marked differential expression in the segregants (p < 0.0001). [file 1471-2105-9-244-S7.pdf]
